# A Web-Based, Mail-Order Sexually Transmitted Infection Testing Program: Qualitative Analysis of User Feedback

**DOI:** 10.2196/48670

**Published:** 2023-09-11

**Authors:** Abagail Edwards, Aries Nuño, Christopher Kemp, Emily Tillett, Gretchen Armington, Rachel Fink, Matthew M Hamill, Yukari C Manabe

**Affiliations:** 1 Center for Indigenous Health Department of International Health Johns Hopkins Bloomberg School of Public Health Baltimore, MD United States; 2 Department of Medicine Johns Hopkins University Baltimore, MD United States

**Keywords:** sexually transmitted infection, STI, HIV, direct-to-consumer screening and testing, web-based systems, feedback, web-based, user, testing program

## Abstract

**Background:**

The incidence of sexually transmitted infections (STIs) is increasing in the United States. The COVID-19 pandemic resulted in significant reductions in access to health care services, including STI testing and treatment, leading to underreporting of STI cases and a need for alternatives to clinic-based testing. Moreover, concerns around confidentiality, accessibility, and stigma continue to limit access to clinic-based STI testing, particularly for high-priority populations. IWantTheKit (IWTK) is a web-based platform that mails free, confidential, self-administered sample collection kits for testing for gonorrhea, chlamydia (both genital and extragenital sites), and vaginal trichomonas. Individuals visiting the IWTK website may select genital, pharyngeal, and rectal samples for chlamydia and gonorrhea testing. Vaginal samples are tested for trichomoniasis. Self-collected samples are processed in a College of American Pathologists–accredited laboratory, and results are posted to an individual’s secure digital account.

**Objective:**

This study aimed to (1) describe users’ experience with the IWTK service through analysis of routine data and (2) optimize retention among current users and expand reach among high-priority populations by responding to user needs through programmatic and functional changes to the IWTK service.

**Methods:**

Free-text entries were submitted by IWTK users via a confidential “Contact Us” page on the IWTK website from May 17, 2021, to January 31, 2022. All entries were deidentified prior to analysis. Two independent analysts coded these entries using a predefined codebook developed inductively for thematic analysis.

**Results:**

A total of 254 free-text entries were analyzed after removing duplicates and nonsensical entries. Themes emerged regarding the functionality of the website and personal experiences using IWTK’s services. Users’ submissions included requests related to order status, address changes, replacement of old kits, clinical information (eg, treatment options and symptom reports), and reported risk behaviors.

**Conclusions:**

This analysis demonstrates how routine data can be used to propose potential programmatic improvements. IWTK implemented innovations on the website based on the study results to improve users’ experience, including a tracking system for orders, address verification for each order, a physical drop box, additional textual information, direct linkage to care navigation, and printable results. Web-based, mail-order STI testing programs can leverage user feedback to optimize implementation and retention among current users and potentially expand reach among high-priority populations. This analysis is supported by other data that demonstrate how comprehensive support and follow-up care for individuals testing positive are critical components of any self-testing service. Additional formal assessments of the IWTK user experience and efforts to optimize posttesting linkage to care may be needed.

## Introduction

Rates of curable sexually transmitted infections (STIs) have been steadily rising in the United States since 2017, with a temporary decrease in chlamydia cases observed in 2020, followed by an increase again in 2021 [1]. From 2015 through 2019, the incidence of chlamydia and gonorrhea increased by approximately 20% and 50%, respectively [2]. Significant reductions in access to health care, including STI testing and treatment, coinciding with the COVID-19 pandemic, raised concerns about STI underreporting during this time [3]. Clinic-based testing for STIs continues to pose barriers for some individuals due to confidentiality concerns, limited availability and restrictive opening hours, the inconvenience of visiting a clinic, and the anticipated stigma [4-7].

IWantTheKit (IWTK) is one of the few mail-in services offering free self-collection kits for lab testing of STIs in the United States [8,9]. Individuals order swabs for self-collection of genital, pharyngeal, and rectal samples for chlamydia, gonorrhea, and trichomoniasis (vaginal samples only) testing; these samples are then processed using nucleic acid amplification tests from Hologic in a College of American Pathologists–accredited laboratory. Results are posted to a user’s secure, HIPAA (Health Insurance Portability and Accountability Act)-compliant account. Oral fluid tests (OraQuick; OraSure Technologies, Inc) are also mailed out to certain jurisdictions for home testing for HIV. In 2022, the demographics of the 5776 individuals who placed an order with IWTK included the following: 49% Black, 69% assigned female at birth, 4% gender diverse, 10% Hispanic, and 25% aged younger than 25 years. IWTK aims to overcome barriers posed by clinic-based testing and offers a way to facilitate access to STI testing and early diagnosis.

It is important to understand the feedback of users who opt for STI testing through a web-based platform like IWTK to tailor services to their needs. Individuals concerned about stigma, those with limited transportation, or those residing in remote areas are among the predominant users of web-based STI testing services [7,8]. Young adults and adolescents may prefer the discretion of self-collect services, as they may not feel comfortable discussing their sexual health with family or health care providers [10]. Historically marginalized communities, including racial or ethnic minorities and lesbian, gay, bisexual, transgender, and queer individuals, may face similar challenges in addition to stigma and discrimination [11,12]. This analysis aimed to describe users’ reported experiences with IWTK, aiming to optimize reach and retention among existing users.

## Methods

### Procedure

Free-text entries were submitted by IWTK users via a “Contact Us” portal on the IWTK website from May 17, 2021, to January 31, 2022. During this period, IWTK was only available to residents of Alaska, Arizona, and Maryland. Alaskan users submitted their queries elsewhere and are not included in this analysis. All entries were deidentified prior to analysis. Duplicates and nonsensical entries were removed. A codebook was developed by 2 analysts through an inductive thematic analysis process, where themes and codes emerged directly from the data [13]. Once the codebook was finalized and tested, the analysts independently coded entries and resolved discrepant codes through discussion until a consensus was reached.

### Ethical Considerations

The Johns Hopkins Institutional Review Board deemed this analysis exempt (IRB00259766), indicating it involves minimal risk and protects the privacy and confidentiality of participants.

## Results

### Overview

Between May 17, 2021, and January 31, 2022, IWTK processed 2818 orders; 270 (9.6%) free-text entries were submitted during this time. Of these, 254 were available for analysis, with 208 (81.9%) from Maryland, 5 (2.0%) from Arizona, and 41 (16.1%) from other jurisdictions. All entries were in English.

### Order Status

More than one-fifth of users (n=57, 22.4%) who submitted queries inquired about the status of their kit order. This included those who were waiting for their kit to arrive after ordering (“I wanted to know if there is a tracking number for my order”) and those who were waiting for their results to be posted to their secure profile after mailing back their completed kit (“I shipped my vials/tests back earlier this week. I don’t see any update as to whether or not they were received back, so I just wanted to check on this”).

### Address Change

A total of 12 (4.7%) submissions included requests to change their delivery address, often occurring after they had already submitted their order (“I changed the address in my profile after submitting the order. I just want to make sure it ships to the correct address”). At the time of analysis, the user’s address could only be altered through their profile rather than at the time of placing an order.

### Kit Replacement

A total of 19 (7.5%) submissions included requests for a replacement kit. One user wrote the following:

I ordered a kit previously, but it was lost during a move, so I wasn't able to complete it. How can I order a new kit?

IWTK only allows new orders to be placed once previous orders have been completed (returned). Users with incomplete kits, either due to their own errors (eg, losing the kit during a move, damaging it, or exceeding the return time frame) or due to an error on the part of IWTK (eg, incorrect swabs based on anatomical site, wrong kit language, or the portal indicating the need to return the HIV self-test), were unable to order replacement kits on the website.

### Clinical Information

In some cases, users wanted to know where they would be able to seek care if they tested positive for an STI (“I’m trying to find out if I test positive, where do I go from there?”) Others were interested in printing out their results from IWTK to show their provider (“I am trying to send my results to my provider so I can get treatment for an STI”). Most submissions regarding clinical information were proactive (eg, information was requested prior to receiving results) rather than reactive (eg, information was requested after receiving results). Overall, 41 (16.1%) submissions were related to users looking for clinical information.

### Disclosure of Reasons for Seeking STI Self-Testing Services

Some users (n=79, 31.1%) used the “Contact Us” page to disclose their reasons for seeking STI testing services, using a tone similar to that of an online forum. We divided their reports into three categories: (1) lack of health insurance, (2) complaints of symptoms, and (3) exposure to an STI or having multiple partners. These subthemes were not mutually exclusive; 2 users reported more than 1 of the subthemes in their submissions. One user wrote the following:

I’m interested in getting the kit because recently my insurance policy got taken away from me and I’ve been having sex with 2 people and my vagina has been a little off but I can’t go to any doctor’s office without insurance.

## Discussion

### Principal Findings

We used deidentified free-text submissions on the IWTK website to offer insights into the IWTK experience for STI testing. IWTK recently implemented innovations to address issues raised in submission queries, including the following: (1) a tracking system for return orders to provide updates on order status; (2) physical address verification for each order to prevent postorder changes; (3) a physical drop box for kit returns in Maryland to promote returns. To address clinical submissions and reasons for seeking testing, the following were implemented: (1) additional textual information on the results screen, instructing users what to do if they test positive; (2) a direct link to care navigation; and (3) printable result sheets. Individuals who use the submission portal to request or report clinical information and disclose sexual behaviors may benefit from additional information and resources to improve their experience. Although the average response time for all queries is 1 business day, an automatic reply directing users to the IWTK “services and resources” tab may enhance user experience.

### Conclusions

This analysis represents an efficient use of routine data for programmatic improvements. Our results align with previous studies suggesting that providing additional support and follow-up care for individuals testing positive are critical components of any self-testing service [14]. Digital supports, in particular, have been deemed broadly feasible, acceptable, and preferable; they have been shown to increase HIV self-test uptake among priority populations [15].

### Limitations

Our findings are limited by the “Contact Us” portal’s focus on specific queries rather than a comprehensive user assessment. This analysis may not have captured all user perspectives. Individuals who used the “Contact Us” submission portal may not have accurately represented the overall IWTK user population. Further, only English language queries were received, potentially excluding individuals unable to read or write in English.

### Future Implications

IWTK is now available in additional states (Kansas, Oklahoma, New Mexico, Nevada, Utah, North Dakota, South Dakota, and Nebraska), and the website is available in Spanish. Future work could include analysis of the “Contact Us” data from a wider geographical area. Other web-based, mail-order STI testing programs could similarly leverage user feedback to optimize implementation and expand reach among high-priority populations. The IWTK platform has been previously used to survey users [16]. Additional formal assessments of the IWTK user experience and efforts to optimize posttesting linkage to care may be required.

## Abbreviations

HIPAA: Health Insurance Portability and Accountability Act
IWTK: IWantTheKit
STI: sexually transmitted infection

## Data Availability

The data sets generated and analyzed during this study are available from the corresponding author upon reasonable request.
